# On, With, By—Advancing Transgender Health Research and Clinical Practice

**DOI:** 10.1089/heq.2022.0146

**Published:** 2023-03-03

**Authors:** Carl G. Streed, Jacob E. Perlson, Matthew P. Abrams, Elle Lett

**Affiliations:** ^1^ Section of General Internal Medicine, Department of Medicine, Boston University Chobanian and Avedisian School of Medicine, Boston, Massachusetts, USA.; ^2^ Center for Transgender Medicine and Surgery, Boston Medical Center, Boston, Massachusetts, USA.; ^3^ The Fenway Institute, Boston, Massachusetts, USA.; ^4^ Department of Psychiatry, Columbia University, New York, New York, USA.; ^5^ Center for Emergency Care Policy and Research, Perelman School of Medicine, University of Pennsylvania, Philadelphia, Pennsylvania, USA.; ^6^ Penn Medicine Center for Digital Health, Perelman School of Medicine, University of Pennsylvania, Philadelphia, Pennsylvania, USA.; ^7^ Center for Applied Transgender Studies, Chicago, Illinois, USA.; ^8^ Computational Health Informatics Program, Boston Children's Hospital, Boston, Massachusetts, USA.; ^9^ Perelman School of Medicine, University of Pennsylvania, Philadelphia, Pennsylvania, USA.

**Keywords:** clinical care, community-led research, health equity, transgender and gender diverse

## Abstract

To advance the fields of transgender health research and clinical care and center trans-led scholarship, there must be an acknowledgment of the consolidated power in cisgender hands and the subsequent need to redistribute such power to trans experts and burgeoning trans leaders. To redress the social structures that cause harm and limit opportunities for trans persons to lead, current cisgender leaders can take actions including deferring opportunities to trans persons to ensure a redistribution of power and resources to trans experts. This article presents necessary steps to recruit, collaborate, and elevate trans experts.

## Introduction

Trans scholars represent a diverse, resilient, and vibrant population that is forced to overcome extensive systemic stigma and discrimination to exist in society, let alone thrive in academia. Throughout this piece we use the term *trans* in the most inclusive way possible; representing all individuals who identify with a gender different than the one associated with the sex they were assigned at birth, including, but not limited to, transgender men and women, and nonbinary, gender nonconforming, and agender individuals. Despite numerous barriers to academic success and career advancement,^1^ ∼1.2% of matriculating allopathic medical students in 2021 were trans according to the Association of American Medical Colleges, up from 0.7% in 2018.^2^ This does not take into account health researchers and other health care professions.^3^ Despite increasing numbers of trans experts in health research and the clinical care of trans populations, these fields remain dominated and dictated by cisgender persons and cis-centric perspectives of health and well-being.

Calls to share resources, center trans-led scholarship, and redistribute power^4^ that is often acquired and maintained by cis-privilege have been met with cisgender fragility, a tendency to engage in defensive posturing and outright denial, minimizing concerns raised by trans communities while overemphasizing good intentions.^5^ Expanding on this point, cisgender researchers and clinicians are raised, trained, and supported in an environment that protects them from gender-identity-based stress and rewards them for their assumed detached objectivity in caring for and studying trans populations.^5^ The language to describe cisgender fragility is rooted in White fragility and has parallel origins in colonization and the resultant consolidation of binary sex and gender.^6,7^ The breadth and depth of emerging scholarship on cisgender fragility are beyond the scope of this article.^5^

To advance the fields of trans health research and clinical care, there must be an acknowledgment of the power consolidated in cisgender hands, an ongoing multipronged approach to training and elevating trans persons in the fields of health care and health research, and the subsequent redistribution of power to trans experts and burgeoning trans leaders.

## Historical Review of Cis-Centric Research and Clinical Leadership

The fields of trans health research and clinical care have evolved and grown considerably since the early-1900s and reveal persistent limitations due to a predominately cisgender perspective. The founding of the Institute for Sexual Research (*Institut für Sexualwissenschaft*) in 1919 by Dr. Magnus Hirschfeld represented an advance in moving trans persons off the front page of salacious newsstands and into the realm of scientific inquiry.^8^ Concurrently, clinicians began to develop medical and surgical interventions (hormone therapy, gender-affirmation surgery, etc.). Subsequently, dedicated centers for trans medical and surgical care began to emerge in the 1950s.^9^

These centers employed onerous invasive approaches to gender-affirming care, which would today be described as gatekeeping; one early leader in gender-affirming surgical care had nearly 1200 applications for gender-affirming surgeries, but only performed 23 (7 transgender women and 16 transgender men) between 1966 and 1972.^9^ This approach to surgical care was evaluated by measuring the “job, educational, marital, and domiciliary stability” of trans persons who had received surgery and reportedly found no “objective advantage in terms of social rehabilitation.”^10^ Beyond inappropriate research methodologies,^9^ researchers failed to acknowledge the role of systemic discrimination and gender nonaffirmation on the well-being of trans persons. Instead, researchers focused on “social rehabilitation” that centers cisgender identities as the ideal and only valid gender experience. Essentially, trans persons were still seen and treated as “other” in clinical and research enterprises.

Even today, the often cost-prohibitive mental health consultations that trans persons require before being eligible for gender-affirming surgeries could be seen as gatekeeping of gender-affirmation procedures. This gatekeeping may also differentially impact trans persons of color, as ethnoracial inequities in access to gender-affirming mental health care have been demonstrated.^11^ Although there has been a shift toward more competent and appropriate care that is patient-centered and gender-affirming, the majority of research and clinical care remains rooted in a deficits-centered approach.^12^

The 2016 call by the National Institute for Minority Health and Health Disparities designating gender minority populations as a health disparity population of interest concretizes this deficits-centered approach to trans health research and clinical care. As research focused on deficit and illness is more likely to receive funding,^13^ cisgender researchers may feel less compelled to design studies that more fully explore the varied experiences and outcomes of trans persons. Cisgender clinicians applying a biomedical lens are likely to pathologize or only capture adverse factors related to trans experiences.

However, strength-based approaches that leverage the intrinsic strength and knowledge of trans and intersecting communities are an important and neglected opportunity for achieving health equity.^14^ Trans-led research would more accurately reflect the complexity and resilience of trans communities, and promote their process for self-determination, self-compassion, and joy.^4^ Although established researchers may be willing to engage with trans persons and move toward a collaborative community-based research enterprise,^4^ we are advocating for the next step in the evolution of trans health research and clinical care: trans experts leading.

## Lessons from the Disability Rights Movement

Evolution in research and clinical care are not unique to trans issues. The imperative for trans representation and leadership in research and clinical care mirrors the historical and contemporary disability rights movement. One particular lesson from disability advocates is that although meaningful representation within decision-making bodies is critical, inclusion does not guarantee unjust structures will be redressed.^15^ Borrowing from the advocacy efforts of persons with disabilities, it is necessary that we transition research and clinical care for trans individuals from on them to with them and ultimately *by* them.^16^ This transition ensures that clinical care and research are not conducted strictly from a singular privileged perspective.

Given the obvious existence of trans persons with expertise in relevant research and clinical disciplines, we recommend similar transitions of power occur in trans-related fields, moving from the perspective of cisgender clinicians and researchers to a multitude of perspectives with trans experts. Specifically, those of us who are cisgender in research and clinical fields should begin to anchor our contributions in the field in redressing the social structures that cause harm and limit opportunities for trans persons to lead. This requires that cisgender colleagues become sponsors for trans scholars and defer resources and opportunities to them.^17^

## Recommendations

To ensure a redistribution of power and resources in the hands of cisgender researchers and clinician leaders to trans experts, cisgender persons can take necessary steps to recruit, collaborate, and elevate trans experts (Table 1). The underlying assumption is one that is common among many health equity scholars^18^ and research paradigms;^19^ specifically, that members of oppressed and excluded groups have experiential knowledge and insider perspective that is vital for achieving (health) equity for that group. Put another way, the lived experiences of trans scholars are part of their “training” in transgender health equity.

**Table 1.** Steps to Redistribute Power and Resources to Transgender and Gender Diverse Experts

Steps	Key components	Examples
Self-reflection	Identify domains where cis-privilege is personally experienced. Acknowledge feelings of defensiveness as manifestations of cisgender fragility. Identify potential elements of extraction from trans communities without commensurate redistribution.	-Actively reflect on personal cis-privilege when beginning a new research project and/or entering a collaboration. This can be in the form of a reflective document for each project. -Be able to articulate a clear reason why you are doing this research project, what biases your positionality *will* foster, and what strengths you have that make you suited for this specific research project.
Pipeline development	Encourage, support, and uplift trans persons in research and clinical care. Design grants and programs to support trans persons.	-Create training programs and opportunities for trans persons. -Connect trans trainees and faculty (with explicit approval of all parties). -Include capacity building in clinical and research initiatives (e.g., funded fellowships).
Sponsorship	Provide mentorship to trans trainees. Actively provide resources with and create opportunities for emerging trans experts.	-Match trans trainees to peers with proven track records of supportive mentorship and sponsorship. -Be aware of trans experts and suggest them for opportunities and initiatives for which you are privy.
Collaboration	Engage in bidirectional collaborations with trans experts.	-Reach out to trans experts to discuss research initiatives, such as NIH request for proposals that have a clear connection to trans issues. Provide support if trans expert is equipped and interested in pursuing research projects. -Ideally, all research on trans populations will be community-informed and include trans collaborators with rich nontokenizing roles.
Amplification	Acknowledge and cite trans experts in publications, projects, and initiatives.	-Read publications by trans experts and save them in citation managers to be easily accessed for citation. -When given the opportunity to discuss trans issues explicitly name trans experts doing the work being discussed.
Sharing power	Redirect resources and opportunities to trans experts (suggested panel participants, advisory boards, etc.).	-Decline invitations to speak on panels regarding trans issues if you are not trans. -Explicitly connect trans persons to opportunities you have appropriately turned down.
Redistributing resources	Create steps to transition power, authority, and resources to trans experts (e.g., stepping down as editor in chief and elevating trans clinician to chief medical officers).	-Develop a 1- and 5-year succession plan to prepare and elevate trans experts. -Redirect leadership offers to trans experts with explicit approval of all parties.
Safety	Ensure safe environments for trans experts to conduct their work.	-Institutional support for inclusive environments (i.e., explicit reporting guidelines for transphobia and inclusive language trainings). -Financial and institutional support for safety for trans experts in response to politically driven threats of violence (i.e., funding for late-night transport and media personnel to help release statements in support of trans scholars targeted by private citizens or political movements).
Accountability	Be responsive and receptive to critique from trans communities inside and outside of academia.	-Outline a dissemination plan in conjunction with trans community members and trans scholars. -Clearly articulate how the proposed research will benefit trans communities.

This assumption is not absolute nor sufficient; not every individual trans scholar studying health will automatically be suited for or desire to be a trans health equity researcher. Trans scholars may choose to apply their considerable talents to other aspects of health, where increasing diversity will benefit health care broadly. Similarly, trans individuals alone are not sufficient to redress the cisnormative practices of health care and society broadly, and require the allyship of cisgender clinicians to be successful.

However, to achieve more robust incorporation of trans perspectives in trans clinical care and research, cisgender clinicians and researchers can start by active self-reflective practices to recognize and evaluate the power they have in the field. This involves acknowledging their cisgender privilege is rooted in being raised, trained, and supported without gender-identity-specific stressors and subsequently rewarded for their perceived objectivity in entering trans fields. To ensure trans trainees are supported in their endeavors to pursue careers in health research and clinical care, cisgender leaders should develop and support a pipeline of trans experts, especially those from historically and contemporarily excluded ethnoracial groups.

Although there are an increasing number of trans clinicians and researchers, the small ranks of trans experts likely reflects the ways in which gatekeeping and gender-identity-specific stressors from youth through adulthood have impacted trans enrollment in and completion of graduate studies in relevant fields. Expanding and strengthening the pipeline for trans experts would also make sure no trans expert is burdened with the expectation or reality of being the sole expert in their field as has been the experience of other experts from marginalized backgrounds. Through sponsorship of and dialogue with trans experts, cisgender experts can establish and maintain bidirectional collaborations with trans experts that ensure trans experts are included and provide a more equitable space for cisgender persons in future work.

The redistribution of power does not mean blind deference but rather requires a back-and-forth dialogue that challenges assumptions, revises and refines hypotheses, and is rooted in the exchange of ideas that facilitates high-quality science and competent care. This process is not expected to be comfortable but rather one that fosters growth of all involved in progressing the health and well-being of trans persons and communities. Concurrently, amplification and dissemination practices within academia will ensure the recognition and centering of trans persons and their work.

As an example, through our own writing process as cisgender and transgender coauthors we sought to (1) address issues and concerns voiced by trans persons, (2) further (rather than recapitulate) the discourse regarding trans health research and clinical care, and (3) provide actionable recommendations that cisgender leaders can implement with trans trainees and experts to create opportunities in the field. This also entails sharing resources with and creating opportunities for trans trainees and experts. Consequently, cisgender researchers and clinical leaders should be prepared to redirect invitations to be on panels, advisory boards, and committees to trans experts and trans persons with relevant experience.

A caveat to such redirection is the recognition of how these opportunities are typically not financially supported and potentially tokenizing. Rather than assume trans experts would not find value in these opportunities, cisgender researchers and clinical leaders should identify additional means of compensation, financial and otherwise. In addition, in today's climate where trans health and bodies are highly politicized, it is imperative that additional steps be taken to ensure the safety of trans individuals who may be targeted in response to their scholarship, activism, clinical work, and existence.

## Conclusion

Ultimately, cisgender leadership can recognize their consolidated power and take steps to redistribute it. Cisgender leaders should prepare timelines for stepping down from positions of power, including editorial boards, advisory committees, and leadership roles in various organizations related to trans health and clinical care and play more supportive, advisory, and mentorship roles. These steps will result in the centering and elevation of trans experts in their respective fields and strengthen opportunities for all persons dedicated to advancing the health and well-being of trans persons and communities.

## Positionality Statement

The authorship team includes two White, gay, cisgender men (first and second author), one queer, cisgender man, and one Black, transgender woman (senior author). This team was purposely selected to allow cisgender individuals to lead the articulation of practices that allow cisgender scholar-advocates in transgender health research to be accountable and equitable allies. We believe that part of the responsibility of cisgender scholars in this field is to call-in each other and offset the labor of dismantling cisnormativity from trans individuals. Through challenging and recurrent conversations among the authorship team we executed the process of bidirectional feedback and reflection that we advocate for in this piece.
